# A More Comprehensive Approach to the Neuroprotective Potential of Long-Chain Polyunsaturated Fatty Acids in Preterm Infants Is Needed—Should We Consider Maternal Diet and the n-6:n-3 Fatty Acid Ratio?

**DOI:** 10.3389/fped.2019.00533

**Published:** 2020-01-10

**Authors:** Susanna Klevebro, Sandra E. Juul, Thomas R. Wood

**Affiliations:** ^1^Department of Clinical Science and Education, Stockholm South General Hospital, Karolinska Institutet, Stockholm, Sweden; ^2^Division of Neonatology, Department of Pediatrics, University of Washington, Seattle, WA, United States

**Keywords:** preterm infant, polyunsaturated fatty acids, docosahexaenoic acid, arachidonic acid, neurodevelopment

## Abstract

There is growing evidence that long-chain polyunsaturated fatty acids (LCPUFAs) are of importance for normal brain development. Adequate supply of LCPUFAs may be particularly important for preterm infants, because the third trimester is an important period of brain growth and accumulation of arachidonic acid (n-6 LCPUFA) and docosahexaenoic acid (n-3 LCPUFA). Fatty acids from the n-6 and n-3 series, particularly, have important functions in the brain as well as in the immune system, and their absolute and relative intakes may alter both the risk of impaired neurodevelopment and response to injury. This narrative review focuses on the potential importance of the n-6:n-3 fatty acid ratio in preterm brain development. Randomized trials of post-natal LCPUFA supplementation in preterm infants are presented. Pre-clinical evidence, results from observational studies in preterm infants as well as studies in term infants and evidence related to maternal diet during pregnancy, focusing on the n-6:n-3 fatty acid ratio, are also summarized. Two randomized trials in preterm infants have compared different ratios of arachidonic acid and docosahexaenoic acid intakes. Most of the other studies in preterm infants have compared formula supplemented with arachidonic acid and docosahexaenoic acid to un-supplemented formula. No trial has had a comprehensive approach to differences in total intake of both n-6 and n-3 fatty acids during a longer period of neurodevelopment. The results from preclinical and clinical studies indicate that intake of LCPUFAs during pregnancy and post-natal development is of importance for neurodevelopment and neuroprotection in preterm infants, but the interplay between fatty acids and their metabolites is complex. The best clinical approach to LCPUFA supplementation and n-6 to n-3 fatty acid ratio is still far from evident, and requires in-depth future studies that investigate specific fatty acid supplementation in the context of other fatty acids in the diet.

## Introduction

**Figure 1:**
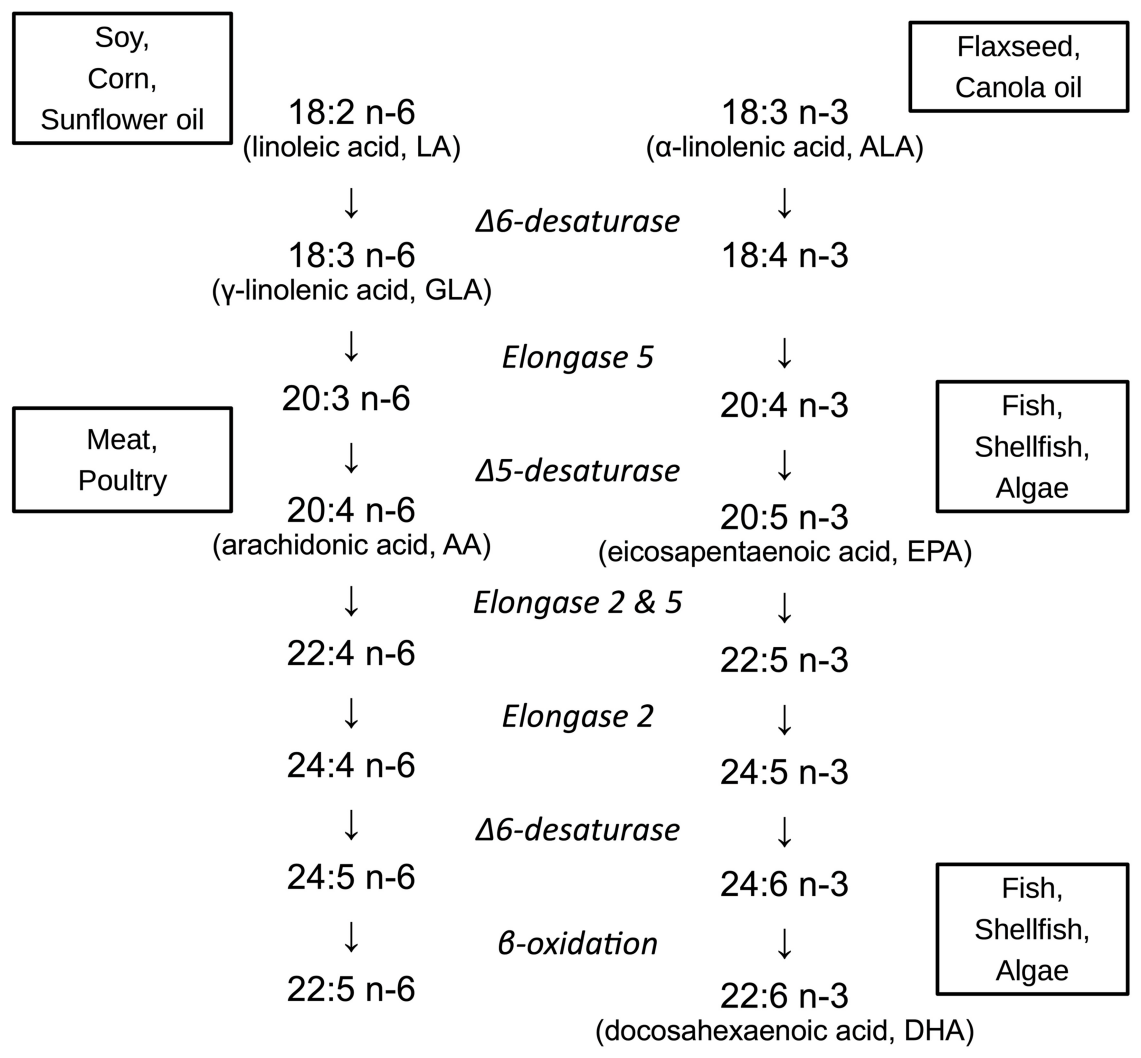
Metabolism and major food sources of important n-6 and n-3 fatty acids. Linoleic acid (LA, 18:2 n-6) is primarily obtained from vegetable oils (soy, corn, and sunflower oils). α-linolenic acid (ALA, 18:3 n-3) is also derived from plant-based oils, such as canola (rapeseed) and flaxseed. Arachidonic acid (AA, 20:4 n-6) derives from animal fats, particularly poultry. Eicosapentaenoic acid (EPA, 20:5 n-3) and docosahexaenoic acid (DHA, 22:6 n-3) are largely obtained from fish, shellfish, and algae.

Infants born preterm are at a high risk of neurodevelopmental disabilities (1–3), with cognitive impairment ranging from 20% in late preterm infants to 64% in extremely preterm infants, of which 34% have moderate or severe impairment (3, 4). The last trimester of pregnancy is an important period of rapid brain growth, during which there is a high susceptibility to brain injury (5–8). It is also a period when the availability of sufficient amounts of long chain polyunsaturated fatty acids (LCPUFAs) is essential to brain development (9). The LCPUFAs in the n-6 and n-3 series have different properties and functions, and to some extent can be synthesized from shorter PUFAs in the diet. For instance, delta 6 desaturase is a key regulatory enzyme needed for the conversion of the n-6 PUFA linoleic acid (LA) to arachidonic acid (AA), and is employed twice for the conversion of the n-3 PUFA α-linolenic acid (ALA) to docosahexaenoic acid (DHA) (10, 11). However, in general LCPUFAs cannot be synthesized *de novo* in sufficient quantities for the developing brain (12, 13). Figure 1 demonstrates the metabolism of the n-6 and n-3 fatty acids.

**Figure 2:**
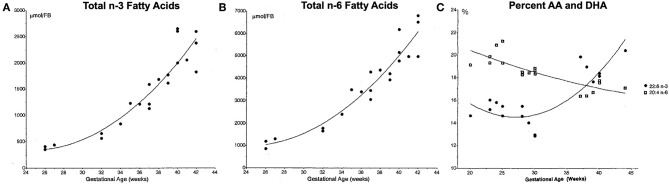
Total n-3 **(A)** and n-6 **(B)** fatty acid forebrain (FB) content, and relative content of docosahexenoic acid (DHA) and arachidonic acid (AA) **(C)**. Fatty acid content measured in post-mortem samples from infants who died soon after birth due to causes not related to the central nervous system. Relative percentages of DHA (22:6 n-3, black circles) and AA (20:4 n-6) in the brain over the final 20 weeks of gestation shows a relative increase in DHA such that the two exist in a ratio of roughly 1:1 at 40 weeks. Reproduced with permission from Martinez (14).

AA and DHA are the most abundant LCPUFAs in the brain. During the last trimester of pregnancy the absolute content of AA and both the absolute and relative content of DHA in the brain increases rapidly (Figure 2). Accumulation continues the first years of post-natal life although the accretion rate slows down (14–17). When preterm birth occurs the supply of LCPUFAs across the placenta is interrupted during a critical period of brain development, which may increase the susceptibility of the brain to both injury and developmental abnormalities. AA and DHA have important functions as components of phospholipids in the plasma membranes of neural cells, including influencing membrane fluidity, which modulates the function of receptors, transporters and membrane-bound enzymes. For instance, both DHA and AA are important for the development of synaptic processes (18, 19). Studies in rats have demonstrated that n-3 fatty acid intake and n-6:n-3 ratio affect expression of genes controlling synaptic plasticity, signal transduction and energy metabolism (20, 21). AA also has specific importance for the structure and function of the endothelial cell, which might be important in vascular regulation, affecting the severity and pattern of preterm brain injury (22). LCPUFAs can also alter membrane and neuronal function, particularly in response to injury. For instance, when the n-6 LA is incorporated into the cell membrane, it becomes highly-susceptible to peroxidation and subsequent ferroptotic cell death (23, 24). AA is also susceptible to peroxidation, and increased concentrations of both LA and AA have been demonstrated to accelerate cell death (25).

**Figure 3:**
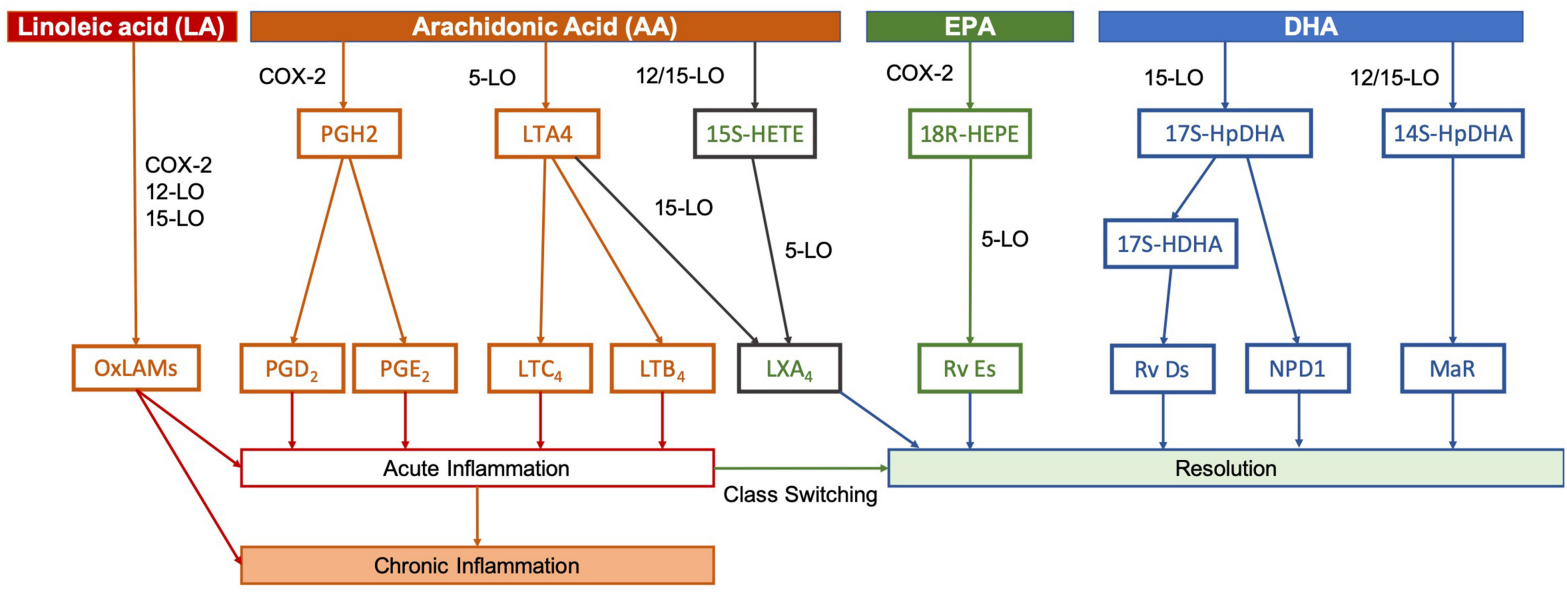
Pathways of lipid mediator production. In peripheral or systemic injuries or infections, phospholipases act on membrane phospholipids to release these PUFAs, whereupon they are metabolized by lipoxygenases (LO) LO-5, LO-12, and LO-15, as well as cyclo-oxygenase-2 (COX-2) into the various lipid mediators. Initially, release of pro-inflammatory mediators, such as prostaglandins (PGs) and leukotrienes (LTs) derived from AA promote the recruitment of neutrophils and initiate the inflammatory process. These pathways are tightly-linked to the process of preterm birth and neonatal inflammatory or hypoxic-ischemic brain injury. Hours to days after an initial inflammatory insult, resolution and healing occurs. Specialized pro-resolving mediators signal this switch, which includes lipoxins (LXs), resolvins (Rvs), (neuro)protectins (NPD/PDs), and maresins (MaRs). Lipoxins, such as LXA4 are derived from AA, but the majority of specialized pro-resolving mediators are produced using EPA and DHA as precursors. Conversely, LA is able to compete with AA, EPA, and DHA for certain COX-2, 12-LO, and 15-LO, resulting in oxidized linoleic acid metabolites (OxLAMs), such as 9- and 13-hydroxy-octadecadienoic acid (9- and 13-HODE) and 9- and 13-oxo-octadecadienoic acid (9- and 13-oxoODE). Adapted from Serhan and Petasis (31).

The relative availability of different LCPUFAs in the face of premature birth or brain injury is likely to alter subsequent responses and may explain epidemiological and preclinical evidence suggesting that n-3 LCPUFAs, such as DHA are neuroprotective (26–28). Inflammation appears to play an important causal role in preterm birth, with ongoing inflammatory responses evident in the post-natal period (29). Inflammatory mechanisms also play a role in altered cerebral development (30). After an initial inflammatory exposure, acute inflammation is intended to be a protective process in two stages—initiation and resolution—and much of the signaling is undertaken by lipid mediators that are the products of LCPUFAs (Figure 3).

Leukotrienes and prostaglandins are produced from AA and play a critical role in initiation of an acute inflammatory response. These pathways are tightly linked to the process of preterm birth and neonatal inflammatory or hypoxic-ischemic brain injury. For instance, increased prostaglandin E2 mediates premature cervical ripening in both lipopolysaccharide-induced murine premature birth and estradiol-induced premature birth in sheep (32, 33). Prostaglandin E2 in the cerebrospinal fluid is associated with the degree of brain injury in asphyxiated term newborns (34), and elevated prostaglandin E2 is seen in the amniotic fluid of infants born preterm (35).

The switch to resolution after the acute inflammatory response is signaled by specialized pro-resolving mediators, lipoxins, resolvins, (neuro)protectins, and maresins. Lipoxins, such as lipoxin A4 are derived from AA, but the majority of the pro-resolving mediators are produced using the n-3 LCPUFAs eicosapentaenoic acid (EPA) and DHA as precursors (31). The n-6 LA can also form pro-inflammatory bioactive oxidized linoleic acid metabolites (36). The production of lipid mediators is catalyzed by a specific set of lipoxygenases (LO-5, LO-12, and LO-15), as well as cyclo-oxygenase-2. Therefore, the relative levels of each mediator may be influenced by the availability of each fatty acid precursor (37–39).

Human milk is the most important source of post-natal fatty acid intake for preterm infants. Human milk contains ~12–26% n-6 fatty acids and 0.8–3.6% n-3 fatty acids depending on the maternal diet (40). Preterm formulas historically did not contain AA and DHA, and assumed that the infant would endogenously synthesize AA (from LA), and EPA and DHA (from ALA). However, additional exogenous LCPUFAs may be required due to inadequate capacity to synthesize these fatty acids endogenously (41). A number of trials have examined the effects of additional AA and DHA both for the mother and in formula of infants born prematurely. As the availability of both n-6 and n-3 LCPUFAs is critical to brain development, and may influence how the brain responds to injury, the objective of this narrative review was to provide an overview of the preventive as well as interventional role of LCPUFAs for brain development in preterm infants, focusing on the balance between n-6 and n-3 fatty acids, including their context within the overall maternal diet. Randomized trials of post-natal LCPUFA supplementation in preterm infants are presented in detail. Results from observational studies, trials of post-natal supplementation in term infants and studies of maternal diet during pregnancy, focused on the n-6:n-3 fatty acid ratio, are also summarized.

## Randomized Trials of Post-natal Supplementation in Preterm Infants

A Cochrane review from 2016 compared LCPUFA-supplemented formula with unsupplemented formula in preterm infants. Meta-analyses of mental development index (MDI) and psychomotor development index assessed with Bayley Scales of Infant Development at 12 months (four trials) and 18 months (three trials) showed no evidence of effect with low quality of evidence (42). In our review, results from 13 randomized controlled trials (RCTs) of LCPUFA intervention for preterm infants that have reported neurodevelopmental outcomes are presented (Table 1). We discuss the trials included in the Cochrane review (52–62), as well as two trials that compared formulas with different ratios of LCPUFA (43–46), one trial of an LCPUFA supplement added in human milk (47–51), and two trials with subgroups of preterm infants at higher risk of neurodevelopmental impairment (63, 64).

**Table 1:** Randomized trials of post-natal supplementation with LCPUFA to preterm infants (43–64).

**References**	**Study subjects**	**Intervention**	**Ratio n6:n3**	**Ratio AA:DHA**	**Number of infants**	**Primary Outcome**	**Comment**
Alshweki et al. (43)	GA 25–32 weeks and/or BW <1,500 g Mean/median GA not known-−30% of infants GA <30 weeks	Formula with DHA+AA (no other LCPUFAs) from first week to 6 months corrected age	Group A 2:1 Group B 1:1	Group A 2:1 Group B 1:1	60 randomized, 24 vs. 21 evaluated at 24 months CA.	Psychomotor-development evaluated with Brunet Lézine Scale at 24 months CA. Higher mean scores in group A compared to B.	Scores in group B but not group A were lower compared to breast fed infants.
DINO trial (44–46)	GA <33 weeks Median GA 30 weeks	High DHA (vs. standard DHA). Mothers supplemented with DHA, or DHA-supplemented formula was given from start of enteral feeds until term age	High DHA 7.7:1 formula 4.4:1 breast milk Standard DHA 9.5:1 formula 7.4:1 breast milk	High DHA 0.4:1 Standard DHA 1.5:1	657 randomized, 322 vs. 335 analyzed at 18 months	BSID-II at 18 months CA. No differences between groups. Mental development index interacted with sex and BW.	Higher mean MDI in girls and in lower BW infants at 18 months. No differences at 2, 3–5 or 7 years.
Henriksen et al. (47–51)	VLBW <1.5 kg Median GA 28.4 weeks	DHA+AA in soy/MCT oil vs. soy/MCT oil added in human milk from enteral intake of >100 ml/kg/d to discharge	Intervention 5.6:1 Placebo 6.5:1 (calculated)^*^ Supplementation: Intervention 2.8:1 Placebo 8:1	1:1	141 randomized, 50 vs. 55 analyzed at 6 months	Cognitive development at 6 months CA. Higher problem solving scores (Ages and Stages Questionnaire) in intervention group.	No difference between groups at 22 months or 8 years. Higher DHA at discharge associated with higher MDI and sustained attention at 22 months CA.
Clandinin et al. (52)	GA ≤35 weeks Mean GA 29.4/28.8 vs. 29.6 weeks	DHA + AA (Algal/Fish DHA) in formula from within 10 days of starting enteral feeds until 12 months CA	Intervention: preterm 7/6.8 Post-discharge 7.4/7.1 Term 9.1/8.6 Control: preterm 7.8 Post-discharge 8.1 Term 10.4	2:1	361 randomized, 46/59 vs. 54 evaluated at 18 months CA.	Safety and efficacy in growth and development.	BSID-II at 18 months CA demonstrated higher MDI and PDI in supplemented groups compared to control.
Ross preterm lipid study (53)	GA <33 weeks Median GA 30 weeks	DHA + AA from fish/fungi and egg/fish in formula from start of enteral feeds until 12 months CA	Intervention: Preterm 5.8/6.5:1, post-discharge 7.8/8.1:1 Control: Preterm 6.7:1 Post-discharge 8:1	Preterm 1.6:1 post-discharge 2.7:1	470 included, 43 breast feeding, Still on diet at 12 months: Intervention, fish/fungal 89/140 egg/fish 91/143 Control: 91/144	BSID at 12 months, Fagan intelligence test at 6 and 9 months, vocabulary checklist at 9 and 14 months. No general effect between groups.	Improved motor index in lower BW group that followed study protocol.
Fewtrell et al. (54)	BW <1,750 g and GA <37 weeks Median GA 30.3 vs. 30.4 weeks	DHA, EPA + AA in formula from 10 days to discharge	Intervention 11.8:1 Control 15.3:1	1.8:1	195 randomized, 84 vs. 74 evaluated at 18 months	BSID-II at 18 months CA. No sig differences between groups.	Non-significant higher MDI in intervention group in infants <30 weeks GA.
Fewtrell et al. (55, 56)	BW ≤2,000 g and GA <35 weeks Mean GA 31.2 vs. 31.1 weeks	DHA + GLA in formula from randomization (mean 14 days, range 1–50 days) until 9 months CA	Intervention 6.3:1 Control 7.2:1	(GLA + AA):DHA Approx 2:1	236 randomized, 106 vs. 93 evaluated at 18 months.	BSID-II at 18 months CA. No sig differences between groups.	Higher MDI among boys in the intervention group. No general differences at 10 years follow up.
van Wezel-Meijler et al. (57)	BW <1,750 g and GA <34 weeks Mean GA 30.4 weeks	DHA + AA in formula from post-natal day 3–7 until 6 months CA	Not known from publication	2:1	55 randomized, 22 vs. 20 evaluated.	Myelination on MRI at 3 and 12 months CA. No sig differences between groups.	No difference in MDI or PDI on BSID-III at 3, 6, 12, or 24 months CA.
Fang et al. (58)	GA 30–37 weeks Mean GA 30.3 vs. 30.0 weeks	DHA + AA in formula from full feeds (+ weight >2,000 g + PMA >32 weeks)until 6 months CA	Total content not known from publication. Ratio LA:ALA in both intervention and control 10:1	2:1	27 randomized 15 vs. 9 evaluated at 12 months CA.	Visual acuity at 4 and 6 months, BSID at 6 and 12 months CA. Higher MDI and PDI in intervention group.	Small study. Significance by repeated measures ANOVA stated, CI not reported. DHA only 0.05% of total fatty acids.
Carlsson et al. (59–61)	GA <33 weeks Mean GA 28.1 weeks.	DHA + EPA in formula. Enrolled at mean 25 days post-natal age, supplementation until 9 months CA	Intervention: preterm 5.2:1 Post-discharge 6.0:1 Control: Preterm 6.4:1 Post-discharge 6.9:1	No AA	79 randomized, Fagan: 33 vs. 34 evaluated at 12 months. BSID: 27 vs. 27 evaluated.	Fagan test of infant development at 6.5, 9 and 12 months CA demonstrated that the intervention group had increased number of looks and decreased look duration.	Only n-3 fatty acids in supplementation. Term formula used from term. No difference in BSID at 12 months CA.
Carlsson et al. (60, 62)	GA <33 weeks Mean GA 28.1 weeks.	DHA + EPA in formula from post-natal day 2–5 until 2 months CA	Intervention: 8.0:1 Control: 8.8:1	No AA	59 recruited, Fagan: 15 vs. 12 evaluated. BSID: 21 vs. 22 evaluated.	Fagan test of infant development at 12 months CA demonstrated that the intervention group had increased number of looks and decreased look duration.	Only n-3 fatty acids in supplementation. Higher MDI in supplemented group at 12 months CA.
Premie Tots Trial (63)	GA <30 weeks Selected infants with early symptoms of autism spectrum disorder (ASD). Mean GA 27 weeks	Omega 3-6-9 vs. canola oil Included at 18–38 months CA Duration of intervention 90 days	Intervention 0.4:1 Control 2.3:1 (supplementation)	GLA:DHA Approx 0.4:1 (supplementation)	31 randomized, all evaluated in ITT analyzes. Three infants missing outcome data handled using maximum likelihood estimation.	Parent reported ratings of behavior and development before and after intervention. Greater improvement in ratings of BITSEA sub-scale ASD in the intervention group.	Later intervention in select at-risk group.
Dolphin trial (64)	GA <31 weeks: (SGA or IVH >1) GA >30 weeks: HIE 2–3, or IVH >1, or neuroimaging abnormalities	DHA, EPA, AA, choline, uridine, cytidine, B12, iodine and zinc added in all enteral feeds from full feeds until 2 years CA	Approx 0.1:1 (supplementation)	Approx 0.1:1 (supplementation)	59 randomized, 24 + 19 evaluated	BSID-III at 24 months CA. No sig differences between groups.	Preterm and term infants at risk. Multiple interventions. Non-significant higher language and cognitive scores in the intervention group.

* *Calculated n-6:n-3 ratio, addition of 0.5 mL study oil per 100 mL breast milk using the mean fatty acid compositions of human milk and the study oils presented in Henriksen et al. (47)*.

*ALA, α-linolenic acid; AA, arachidonic acid; ASD, BSID, Bayley scales of infant development; CA, corrected age; DHA, docosahexaenoic; EPA, eicosapentaenoic acid; GA, gestational age; GLA, γ-linolenic acid; LA, linoleic acid; LCPUFA, long-chain polyunsaturated fatty acid; MDI, mental development index; PDI, psychomotor development index*.

Two RCTs evaluated the effect of different AA to DHA ratios on neurodevelopment. A study by Alshweki et al. randomized 60 newborns <1,500 g and/or <32 weeks gestational age (GA) to two formulas with different AA contents and a fixed DHA content (around 0.3%), for 1 year. The study demonstrated higher mean scores of psychomotor-development at 24 months corrected age (CA) with an AA: DHA ratio of 2:1 compared to 1:1. The formulas contained no other n-6 or n-3 fatty acids and results might be related to the low content of AA in the 1:1 group. The scores in the 2:1 group were similar to the scores of exclusively breastfed infants (43). Conversely, the DINO trial randomized infants born <33 weeks' gestation to formula with a high DHA content (1%) to normal DHA content (0.3%), with a fixed content of AA (0.4%). The intervention was provided as tuna oil or soy oil given to breastfeeding mothers, and as LCPUFA-enriched formulas, until the infants reached term CA. The groups did not differ in overall Bayley scales of infant development (BSID)-II score at 18 months, but in the high DHA group mean MDI was higher in girls (mean MDI 99.1 vs. 94.4) and in lower BW infants (mean MDI 94.8 vs. 90.0) (44). However, no differences were demonstrated in behavior or attention at 3–5 years of age or intelligence quotient (IQ) at the age of 7 (45, 46).

The other trials we identified compared supplementation with DHA (and AA in most studies) to supplementation with only LA and ALA and no DHA or AA. A Norwegian trial added soy/MCT oil to all enteral intake (mothers' milk or donor human milk) given to very low birth weight infants until discharge, and compared addition of DHA and AA (ratio 1:1) to no addition of DHA and AA. They demonstrated higher problem-solving scores at 6 months, but no structural changes on MRI or differences on cognitive tests at 8 years of age (47–51). A higher DHA in plasma after the intervention was associated with higher MDI and sustained attention at 22 months (48). Results from that study also showed that higher blood levels of DHA at 8 years of age were associated with higher IQ at that age (51).

Two studies continued intervention with AA/DHA supplemented formula until 12 months CA (52, 53). Clandinin et al. randomized infants born before 36 weeks of GA. Low birth weight infants who had received >80% formula intake before term and >100% formula intake after term were evaluated. The results showed that infants who had received supplemented formula had higher MDI and psychomotor development index (52) at 18 months CA. Mean MDI was 87 in a group supplemented with fish-DHA, 83 in a group supplemented with algal-DHA and 77 in the unsupplemented group. The Ross preterm lipid study included infants born before 33 weeks GA. The study did not demonstrate any general effect of supplementation (DHA + AA in formula) from start of enteral feeds until 12 months CA on BSID evaluated at 12 months CA (53). Fewtrell et al. have published results from two trials. The first study included preterm infants born before 37 weeks of GA with a birth weight of <1,750 g and evaluated an intervention of AA, EPA, and DHA until discharge (54). The second study included infants born before 35 weeks of GA with a birth weight ≤2,000 g and evaluated an intervention of γ-linolenic acid and DHA to 9 months CA (55). Neither of the trials demonstrated any statistically significant differences on BSID-II at 18 months CA compared to control formula. Two additional smaller trials presented conflicting results of supplementation with AA and DHA to 6 months CA (57, 58). Fang et al. reported higher MDI and psychomotor development index in the intervention group at 6 and 12 months CA (58), whereas no differences were shown in the study by van Wezel-Meijler et al. at 3, 6, 12, or 24 months CA (57). In most studies a combination of DHA and AA have been used as intervention. Two older trials studied addition of only n-3 LCPUFAs (DHA + EPA) to infants born before 33 weeks GA (59, 62). In the first trial the intervention period was until 9 months CA. No differences in cognitive development at 12 months CA were demonstrated (60). In a later trial, the duration of the intervention was shorter, until 2 months CA, and the supplemented infants had higher MDI at 12 months CA (60). Both trials demonstrated a more mature pattern of visual attention in the intervention group at 12 months CA, and the authors speculated this was related to more rapid information processing (61, 62).

Two studies have evaluated the effects of LCPUFA supplementation on specific preterm populations at higher risk of neurodevelopmental impairment. One study initiated intervention at 18–38 months CA in a selected group of preterm infants born before 30 weeks' gestation who demonstrated early symptoms of autism spectrum disorder (ASD). They demonstrated an effect on parent-reported autism symptoms after 90 days of intervention that included γ-linolenic acid, EPA and DHA (63). Another study provided a combination of DHA, uridine and choline (components of phosphatidylcholine), and micronutrients to preterm and term infants who fulfilled brain injury inclusion criteria that indicated a higher risk of neurodevelopmental impairment. Treatment was given from start of full enteral feeding to 24 months CA (64), and included a dose of DHA equivalent to recommended total daily intake, as well as a very low dose of AA. Of the 59 randomized infants, 66% completed full dose supplementation for 24 months. The results did not achieve significance, although higher cognitive and language scores in the supplemented group at 24 months indicated a possible effect.

## Context of Fatty Acid Supplementation in Preterm Infant Trials

Across all the studies found, the total n-6:n-3 ratio was higher in the control formula compared to the intervention formula in the majority of the studies, but the differences were small. Ratios around 7:1 have been most commonly used, but preterm control formulas had n-6:n-3 ratios ranging between 5:1 and 15:1. The contribution of DHA was 0.2–0.3% of total fatty acids in most of the studies, as high as 1% in two of the studies (44, 47), and 0.5% in one study (54). LA content varied from 0% of total fatty acids in a study by Alshweki et al. that only added AA and DHA in control and intervention formula (43), to around 11% in the studies by Fewtrell et al. (54, 55), and around 20% in the studies by Carlson et al. (59) and Carlson and Werkman (62). As LA is highly-susceptible to peroxidation and mechanistically competes for the same enzymes involved in the metabolism of AA and DHA, variability in LA (and other fatty acid) content in the formula or breast milk of the intervention studies completed so far may provide a significant confounding factor.

Epidemiological studies have also demonstrated associations between LCPUFAs, brain structure and neurodevelopment in preterm infants. In a cohort study of 51 infants born before 36 weeks of GA, AA:DHA ratio was negatively associated with mental, motor development and orientation on BSID-II at 18 months in adjusted analyses (65). Tam et al. showed that higher levels of DHA in erythrocytes in an early post-natal age was associated with reduced risk of IVH and improved language scores at 30–36 months CA among 60 infants born before 32 weeks of GA. They also demonstrated associations between levels of both DHA and LA and diffusivity in specific brain regions (26).

During the initial post-natal days, preterm infants, particularly those born extremely preterm, often rely exclusively on parenteral nutrition. Parenteral lipid composition might therefore be important to reduce post-natal deficiencies of LCPUFAs. No published studies of newer lipid emulsions containing DHA have reported neurodevelopmental outcome (66, 67). Solely parenteral intervention is unlikely to be sufficient to provide preterm infants LCPUFAs at intrauterine rates, but might be important in a select group of infants with a long period without enteral nutrition.

## Fatty Acid Ratio and Neurodevelopment in Term Infants

Studies of term infants have demonstrated some benefit of LCPUFA supplementation, as well as the importance of finding a proper ratio. In the DIAMOND study, term infants were given post-natal supplementation with three different doses of DHA, at a AA:DHA ratio of 2:1, 1:1, and 0.7:1 compared to unsupplemented control formula. The trial was conducted in two different centers (68). One of the centers, with study infants from a population with a low socioeconomic status, performed cognitive testing every 6 months, and demonstrated higher scores in the intervention groups on several tests (69). Positive effects were seen primarily with ratio 2:1 and 1:1. At 6 years verbal IQ was higher when all supplemented groups were compared to the control group. Follow-up at 9 years demonstrated effects on brain structure, function, and neurochemical concentrations. The group with 1:1 supplementation had greater connectivity between pre-frontal and parietal regions, and groups with 2:1 and 1:1 supplementation had greater white matter volume in regions associated with attention and inhibition (70). Results from the other center in the DIAMOND study demonstrated higher MDI, higher emotional regulation scores and higher language scores when all supplemented groups were compared to the control group (71).

## Maternal Diet

Maternal LCPUFA status is affected by dietary intake and fatty acid metabolism related to variability in desaturase genes (40). Polymorphisms in the FADS gene of the mother have been associated with IQ of the child (72), and have also been shown to modify the association between breast milk and IQ (73). In the last century there has been a shift in dietary fatty acid intakes, with a 20-fold increase in intake of vegetable oils without any significant changes in our genes (74–77). The shift in diet is illustrated in a study of US women demonstrating an increase in the LA:ALA ratio in breast milk samples from the 70s and onwards (78). Higher n-6:n-3 ratio is also associated with increased risk of obesity (79), and obesity is related to increased risk of preterm birth (80). One recent study also demonstrated association between low maternal DHA and EPA levels and increased risk of preterm birth (81). The most recent Cochrane review concluded that there is high quality evidence from multiple clinical trials that n-3 LCPUFA supplementation reduces the risk of preterm birth and low birth weight (82). Some differences in neurodevelopment were noted in this review, but the evidence was graded as low to very low quality (82). Trials of LCPUFA supplementation during pregnancy have also not specifically presented results of neurodevelopment in infants born preterm (83–90). Two big cohort studies have demonstrated a negative association between n-6:n-3 ratio of maternal intake during pregnancy and neurodevelopment in term infants (91, 92). Significant interaction between socioeconomic factors, DHA intervention and cognitive outcome have been demonstrated (88, 89). For instance, in the recent KUDOS trial (Kansas University DHA Outcomes Study), DHA supplementation during pregnancy was associated with a number of improved cognitive outcomes, but this effect was lost after adjusting for socioeconomic status (SES) (93). We speculate that diets in low resource settings are more likely to be higher in LA relative to DHA intake, and dietary interventions could have a greater effect in these populations. However, little high-quality evidence exists to examine n-6 and n-3 intakes at the population level. Two studies using data from the National Health and Nutrition Examination Survey (NHANES) found that both EPA/DHA and LA intake increase with increasing education and income-to-poverty ratio (94, 95). However, this data is notoriously inaccurate, with almost two-thirds of female participants reporting dietary intakes that are not physiologically plausible (96, 97). This is particularly relevant considering that the accuracy of dietary reporting decreases with increasing body mass index, and lower SES is associated with higher body mass index, particularly in women (98). As LA intake has steadily increased in recent decades, the baseline intake across all levels of SES may be such that n-6 reduction strategies need to be implemented alongside DHA supplementation in order to reduce oxidized linoleic acid metabolites (39). As higher maternal SES may protect against some of the negative effects of suboptimal n-6:n-3 intake ratios, it is of particular interest to examine whether levels of LA intake and supplementation with DHA have a more pronounced effect in populations with lower SES.

## Discussion

No single LCPUFA acts in isolation, and the associations between brain development, inflammation and injury are complex. Much of the research to date, including many interventional trials, has targeted the addition of supplemental DHA. Many trials have also included the n-6 AA, and a few clinical trials have examined the effect of AA:DHA ratio on neurodevelopment. The RCTs in this review utilized different LCPUFAs, different doses, different durations, and a variety of outcome measures. Difference in cognitive outcome evaluated with some version of BSID at 18 months CA was the most common primary outcome. To achieve 80% power and 95% confidence to detect a 6 point difference in BSID mental developmental index (MDI) between groups with an effect size (ES) of 0.4, ~100 would be needed in each group. Four of the trials evaluated ~100 infants (44, 53–55). In the DINO trial the power calculation was based on a power of 85%, and also included sub group analyses. However, with a more conservative estimate of 85% power to detect a 4 point difference in MDI between controls and infants treated with DHA, 253 infants would be required in each group. The DINO trial and the Norwegian trial investigated effects on attention and behavior using the Strengths and Difficulties Questionnaire (SDQ) and did not demonstrate differences between the groups. In a subset of the DINO trial, 61 infants given a high DHA supplement were compared to 64 infants who had received a standard DHA supplement at the age of 3–5 years (45). In the Norwegian trial 45 infants in the intervention group were compared to 53 infants in the control group at 8 years of age (50). Based on the available data, an intervention including DHA would expect an ES of 0.3 in total SDQ score as well as its subscales (emotional symptoms, conduct problems, hyperactivity-inattention, peer relationship problems, and prosocial behaviors). To detect a meaningful 1.5 point difference in total SDQ score with a standard deviation of 4.5 with 85% power, 235 per group to include comparisons of subscales. This suggests that, with the exception of the DINO trial, the majority of studies have been significantly underpowered to detect differences in developmental outcomes after DHA and/or AA supplementation. So while a lot of both preclinical and clinical evidence indicates the importance of LCPUFAs in neurodevelopment and neuroprotection, the best clinical approach is far from evident.

The timing and duration of the intervention are of importance. None of the studies in this review targeted maternal diet during pregnancy and lactation as well as infant diet during both the neonatal and post-term period. Preclinically, mice fed a diet deficient in n-3 fatty acids during pregnancy and 6 months thereafter had altered synaptic protein expression in the hippocampus and impaired memory at 6 months compared to mice fed and diet adequate in n-3 fatty acids. If the diet was changed at 3 weeks post-natally the results were normalized, but if the diet was changed at 2 or 4 months the content of fatty acids in the brain was normalized but the effect on synaptic protein expression, learning and memory were not (99). Results from the trials in preterm infants as well as the fact that the brain continue to accumulate DHA the first 2 years of life indicate that continued intervention past term age might increase the probability of long term effect.

The selection of infants in the included studies also varied. Some trials examined the effect on brain development in a population that excluded infants with moderate to severe neonatal complications, who are at the greatest risk of developmental impairments. One of the trials targeted a population at high risk of neonatal brain injury (64). Three of the trials indicated greater benefit of LCPUFA supplementation on neurodevelopment in more immature infants (44, 53, 54). Importantly, the studies in this review were conducted in settings with good access to neonatal care, which leaves the possibility that intervention with LCPUFAs might prove to be more beneficial for moderate preterm infants in a low resource setting.

Ensuring consistent and sensitive outcome assessments will be important when determining the effects of additional LCPUFAs. The intervention might also affect different regions of the brain during different periods, which is why the dosing and the timing of the intervention matter to the expected outcome. In the trial by Henriksen et al., a positive result on free-play sessions at 20 months CA in the intervention group suggested that supplementation with AA and DHA might be related to improved attention (48). Better sustained attention at 22 months CA was also associated with DHA status at discharge in that study (48). Studies in term infants have demonstrated better sustained attention at 5 years of age in term infants whose mothers were given DHA supplementation during pregnancy (90) or the first months after birth (100). A population-based cohort study in Spain demonstrated that higher AA:DHA ratio in cord blood was associated with higher risk of subclinical ADHD symptoms at 7 years of age (101). Reviews and meta-analyses of intervention studies typically focus on a global outcome measure, such as BSID at 18 months CA. However, LCPUFA intervention might lead to more subtle effects, and therefore specific cognitive assessment measures, such as attentional capacity, may be more relevant.

Different types of supplementation may specifically benefit different brain regions. For instance, classes of phosphoglycerides in white and gray matter have unique profiles of LCPUFAs (102). Higher levels of DHA in erythrocytes after birth have been associated with increased gray matter volume in preterm infants (103). DHA is highly prevalent in serine phosphoglycerides in the cerebral gray matter, which is important for long-term potentiation, critical to memory formation (104, 105). Similarly, spatial learning and memory was improved in rats given post-natal DHA supplementation, but excess DHA had an adverse effect (106). Unfortunately, however, few studies included specific tests of memory function. In a 10 years follow-up, Fewtrell et al. performed several specific tests of learning and memory. They did not demonstrate any general differences between the randomized groups, but among 39 infants who were fed exclusively formula and no breast milk, infants fed trial formula containing γ-linolenic acid and DHA had better results on some of the cognitive subtests and higher word-pair learning scores (56). Both control and supplemented formula in the DINO trial had a higher content of LA and a higher total n-6:n-3 ratio compared to breast milk from both mothers who received placebo capsules with soy oil and DHA enriched capsules with tuna oil (45). Therefore, it is worth considering the composition of fatty acids in background feeding and what proportion of trial formula and supplement that has been given. For instance, in rats, a maternal diet with similar DHA intake but 10-fold higher LA intake decreased DHA levels in the brain and worsened motor deficits after hypoxic-ischemic brain injury in the late-preterm equivalent rat (107).

As metabolites of primarily n-3 LCPUFAs, the specialized pro-resolving mediators display a wide-range of anti-inflammatory and cytoprotective functions including decreased cytokine and PG/LT release, decreased recruitment of peripheral neutrophils, and class-switching of activated macrophages and microglia (31, 108). All LCPUFAs utilize the same enzymes for conversion to biologically active eicosanoids (36, 109), and the availability of LA, AA, EPA and DHA as substrates affect the formation of pro-inflammatory and pro-resolving mediators (37–39, 110). Neuroprotective effects of exogenous administration of pro-resolving mediators have been demonstrated *in vitro* and in animal studies (111–113). In a mouse model, increased n-3 fatty acids reduced preterm birth, and were also associated with reduced gene expression of pro-inflammatory cytokines as well as increased levels of pro-resolving mediators (114). Increased LA availability might both decrease the production of pro-resolving mediators, and increase the production of oxidized linoleic acid metabolites. Pro-resolving lipid mediators may shorten the times required for cessation of inflammation, possible leading to decreased tissue injury. Experimentally, deficiencies in pro-resolving mediators as well as high levels of oxidized linoleic acid metabolites have been associated with chronic inflammation and acceleration of associated disease processes (115–119). A low n-6:n-3 fatty acid ratio, both in the diet and in the blood, is also associated with an increased risk of adult neurological diseases, including stroke and dementia (120–122).

AA and DHA seem to have antagonistic and synergistic effects in terms of response to injury, and both are crucial for normal brain development. In baboons the levels of AA in the brain were not affected by dietary intake, whereas the levels of DHA were (123). Studies in rats have also shown that the most important determinant of membrane fatty acid composition in the brain was the dietary n-6:n-3 ratio (124). However, the optimal intake of DHA during the neonatal period in humans has not been determined. At high levels of DHA and EPA, AA becomes suppressed (125), and correlation between low levels of AA and poor growth have been demonstrated (126). Rodent models of neonatal brain injury have demonstrated neuroprotective effects of DHA through several potential mechanisms (27, 127, 128). Labrousse et al. demonstrated association between n-3 fatty acid deficiency and increased levels of cytokines (127). Suganuma et al. showed that DHA supplementation during pregnancy reduced oxidative injury and apoptosis in neurons in a model of neonatal hypoxic ischemic encephalopathy (128). Substrate competition and enzyme inhibition might limit the conversion of ALA and EPA to DHA, especially if there is an abundance of LA (129, 130). Makrides et al. demonstrated that infant formula with the same content of LA but lower ratio of LA:ALA resulted in higher levels of n-3 fatty acids (131). Importantly, the capacity for enzymatic conversion of ALA to DHA is likely to be insufficient relative to the needs in preterm infants (13, 132).

Though DHA is essential for normal neurodevelopment, there is likely to be an upper of limit of benefit, depending on the specific context. In the DIAMOND study of term infants, the highest DHA intake—relative to the intake of AA (AA to DHA ratio of 1:2)—was not associated with positive effects on several of the outcome measures whereas the groups with AA to DHA ratios of 2:1 or 1:1 demonstrated positive results (69). In the DINO trial, however, preterm infants with high DHA intake showed better results in some sub-groups. This illustrates the need for a comprehensive approach to LCPUFA supplementation considering both the levels and ratios of multiple fatty acids and the period of supplementation. As a rapid relative accumulation of AA in the brain is not seen until term-equivalent age (Figure 2C), the need for DHA supplementation would appear to be higher during the preterm period, with an increase in AA to DHA ratio being more appropriate after term-equivalent age.

Although the most recent Cochrane review did not show any significant effect of LCPUFA supplementation (42), two other recent systematic reviews, Shulkin et al. (133) and Wang et al. (134) concluded that LCPUFA supplementation in preterm infants has short term neurodevelopmental benefits. Lapillone and Moltu also concluded that there is evidence of short term benefit with a high dose DHA supplement and their review focused on several areas of benefit in preterm development (135). Smith and Rouse published a narrative review in 2017 focused on the role of DHA in preterm infants (136), and Laurizen et al. have written an interesting commentary on AA (137). A comprehensive overview of several fatty acids in infant development was published by Delplanque et al. (40). Our focus has been on the roles of n-6 and n-3 fatty acids in neurodevelopment of preterm infants, combining a discussion focused on the n-6:n-3 fatty acid ratio with a summary of RCTs of post-natal LCPUFA supplementation to preterm infants with neurodevelopmental outcomes.

## Summary

The complex interplay between LCPUFAs, their metabolites, preterm brain development and mechanisms of brain injury is not fully understood. Future research should have a more comprehensive approach to the LCPUFAs and especially examine the potential harm of unbalanced n6:n3 and AA:DHA ratios, including those in the maternal diet. Comparisons of different LCPUFA compositions, resulting in differing n-6 to n-3 ratios by for example limited or high intakes of LA is an area of priority in our view. Maternal diet and genetic activity need be considered in future studies. Other reviews have also concluded that there is a need for studies with large sample sizes that enable subgroup analyses (42). Outcome measures of LCPUFA supplementation to preterm infants need to target cognitive development, attention and behavioral disorders. Given that there is some evidence of effect on attention from previous studies in preterm infants as well from studies of other groups at risk of attention problems, outcome measures focused on attention would be an interesting priority. The neuroprotective potential of LCPUFAs to specific groups who have already suffered intra cerebral insults also needs further evaluation. Utilized correctly, LCPUFAs could prove to be a cost effective and health promoting part of neonatal care.

## Author Contributions

SK performed the literature search, reviewed the studies, and drafted the manuscript. SJ and TW edited and revised the text. SK and TW made the figures. All authors approved the final version.

### Conflict of Interest

The authors declare that the research was conducted in the absence of any commercial or financial relationships that could be construed as a potential conflict of interest.

## Abbreviations

AA: arachidonic acid
ALA: α-linolenic acid
BSID: Bayley scales of infant development
CA: corrected age
DHA: docosahexaenoic
EPA: eicosapentaenoic acid
GA: gestational age
IQ: intelligence quotient
LA: linoleic acid
LCPUFA: long-chain polyunsaturated fatty acid
MDI: mental development index
RCT: randomized controlled trial
SES: socioeconomic status.
